# Impact of hormone receptor status and distant recurrence-free interval on survival benefits from trastuzumab in HER2-positive metastatic breast cancer

**DOI:** 10.1038/s41598-017-00663-1

**Published:** 2017-04-25

**Authors:** Hai-Yuan Yang, Ding Ma, Yi-Rong Liu, Xin Hu, Jian Zhang, Zhong-Hua Wang, Gen-Hong Di, Xi-Chun Hu, Zhi-Ming Shao

**Affiliations:** 1 Department of Breast Surgery, Fudan University Shanghai Cancer Center, Department of Oncology, Shanghai Medical College, Fudan University, Shanghai, 200032 China; 2 Department of Medical oncology, Fudan University Shanghai Cancer Center, Department of Oncology, Shanghai Medical College, Fudan University, Shanghai, 200032 China

## Abstract

We sought to investigate the impact of hormone receptor (HR) status and distant recurrence-free interval (DRFI) on the degree of overall survival (OS) benefit from palliative trastuzumab-containing treatment in HER2-positive metastatic breast cancer (MBC). Here, we retrospectively identified 588 eligible HER2-positive patients with postoperative distant recurrence. DRFI of HR+HER2+ MBC patients (median: 30.7 months, IQR: 18.5–45.9, *P* < 0.001) was significant longer compared with HR−HER2+ patients. Patients were categorized into four subgroups based on HR status and palliative trastuzumab (trast+) received. The most superior outcome was observed in the HR+HER2+trast+ subgroup, with a median OS of 48.3 months. Moreover, DRFI > 24 months is an independent favourable prognostic factor for both HR−HER2+ patients (Hazard Ratio (HzR) = 0.55, 95% CI: 0.39–0.76, *P* < 0.001) and HR+HER2+ patients (HzR = 0.45, 95% CI: 0.32–0.64, *P* *<* *0*.*001*). Upon further analysis of the interaction between trastuzumab and DRFI, the degree of trastuzumab benefits in HR−HER2+ MBC patients remained basically unchanged regardless of DRFI length. Unlikely, the degree in HR+HER2+ MBC patients decreased gradually along with DRFI extending, indicating that trastuzumab failed to translate into an OS benefit for late recurrent (DRFI > 5years) HR+HER2+ MBC patients.

## Introduction

HER2/neu overexpression and/or amplification is observed in approximately 15% to 30% of invasive breast cancers and is associated with a high risk of relapse and poor prognosis. Trastuzumab has dramatically changed the natural history of HER2-positive disease and has therefore become the standard of care for both metastatic and primary HER2-positive breast cancer^1^.

Emerging evidence indicates that clinically defined HER2-positive breast cancer is clinically and biologically heterogeneous. Approximately 50% of HER2-positive disease is also hormone receptor (HR)-positive^2^, and most cases of HR-positive/HER2-positive (HR+HER2+) breast cancers determined by immunohistochemistry fall into the luminal B subtype based on gene expression^3, 4^. Moreover, the site of recurrence and the rang of distant recurrence-free interval (DRFI) differ by HR status within HER2-positive case^5, 6^.

Although HR status clearly defines at least two distinct subtypes of HER2-positive metastatic breast cancer (MBC), trastuzumab-containing palliative treatment has not been fully optimized for HR+HER2+ MBC patients^7^. To date, almost all randomized clinical trials have been designed to combine trastuzumab with chemotherapy regimens, regardless of HR status, except for a few studies that investigated trastuzumab plus endocrine therapy for patients with HR+HER2+ MBC^8^. Clinicians prefer to recommend chemotherapy combined with trastuzumab as a first-line treatment, while endocrine therapy is most often prescribed in sequence rather than as an alternative.

A gene expression-based predictive model has identified a subgroup of HER2-positive primary breast cancer patients with the highest levels of *ESR1* and its associated mRNA who derived no clinical benefit from adjuvant trastuzumab^9^. However, the impact of HR co-expression on the overall survival benefit from a variety of therapies for HER2-positive MBC remains poorly understood^9–11^. DRFI, which is simple to measure, easy to interpret and clinically meaningful, has been recognized as a predictor of trastuzumab resistance and endocrine therapy resistance. Several retrospective studies have suggested that the DRFI partially accounts for the trastuzumab benefit^12^ and overall survival (OS) after metastasis^13^.

Here we report the palliative treatment pattern and clinical outcome of HER2-positive MBC patients in China. In particular, we investigate the impact of HR status on the OS benefit of trastuzumab-containing palliative therapy, and our results further elucidate the magnitude of the trastuzumab benefit interacting with DRFI as a continuous covariate.

## Results

### Patient characteristics

**Figure 1:**
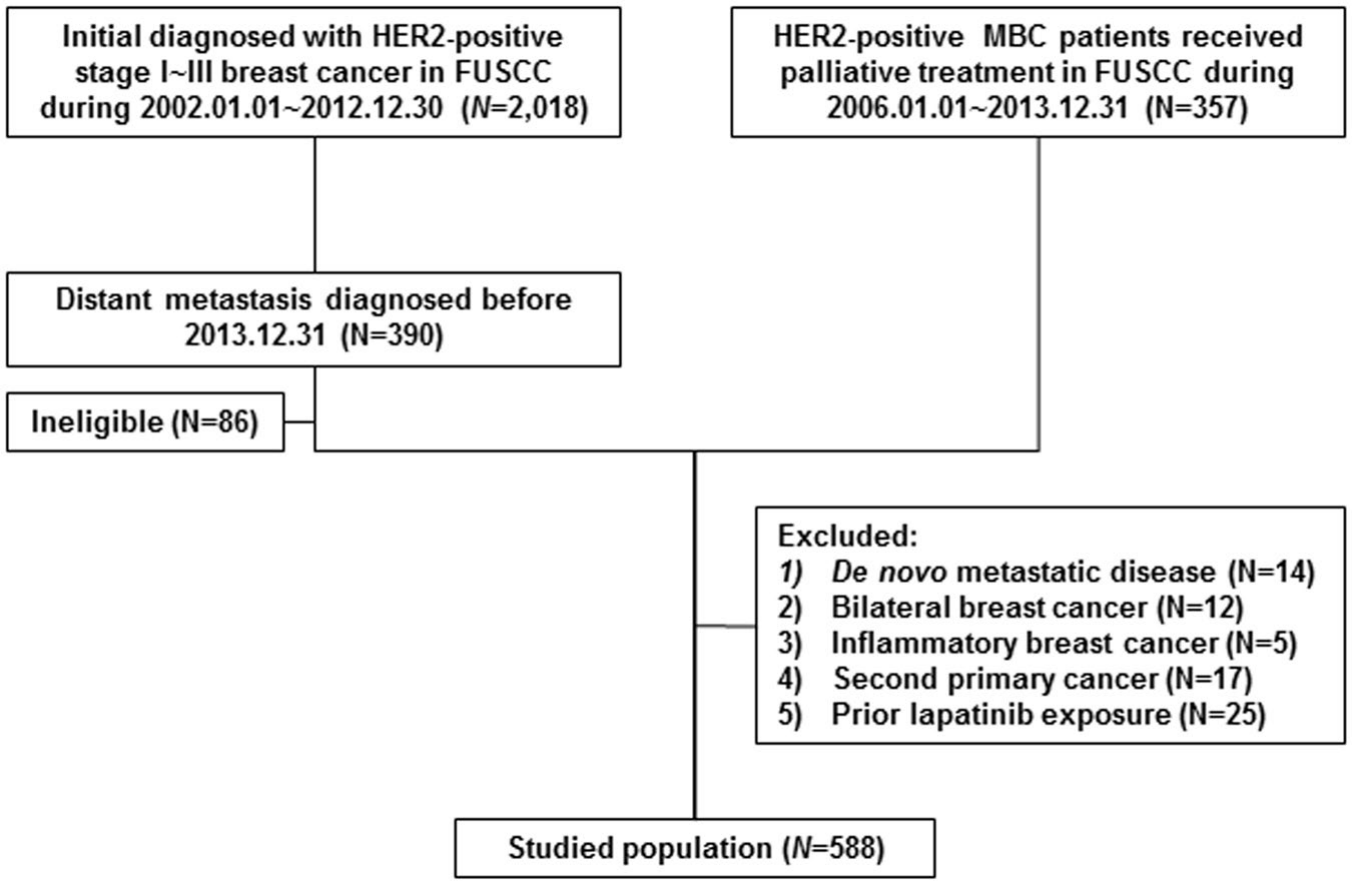
Flowchart of the study population.

In total, 588 eligible HER2-positive MBC patients were retrospectively collected (Fig. 1). Of these, 311 were HR-negative, and 277 were HR-positive. The median follow-up was 24.8 (IQR: 14.0–40.3) months for HR−HER2+ patients and 32.5 (IQR: 29.8–46.9) months for HR+HER2+ patients. Tables 1 and 2 give the patients’ characteristics at the time of primary diagnosis and at the time of metastasis, respectively.

**Table 1:** Patient characteristics at breast cancer diagnosis.

Characteristics	HR−HER2+		HR+HER2+		*P* ^#^
	*N* = 311	%	*N* = 277	%	
Median age (y)	51	—	47	—	
Menopausal status					
Premenopausal	184	59.16	198	71.48	<0.01
Postmenopausal	127	40.84	79	28.52	
Histologic type					0.82
Ductal carcinoma	303	97.43	268	96.75	
Lobular carcinoma	2	0.64	3	1.08	
Other	6	1.93	6	2.17	
Grade					<0.01
I	9	2.89	7	2.53	
II	106	34.08	132	47.65	
III	167	53.70	105	37.91	
Unknown	29	9.32	33	11.91	
LVI					0.44
Yes	181	58.20	147	53.07	
No	95	30.55	97	35.02	
Unknown	35	11.25	33	11.91	
T stage					0.14
1	119	38.26	85	30.69	
2	150	48.23	154	55.60	
3	42	13.50	38	13.72	
N stage					0.87
0	108	34.73	87	31.41	
1	79	25.40	78	28.16	
2	66	21.22	56	20.22	
3	54	17.36	53	19.13	
Unknown	4	1.29	3	1.08	
(Neo) Adjuvant chemotherapy					0.93
Received	305	98.07	271	97.83	
None	6	1.93	6	2.17	
Type of chemotherapy					0.97
Anthracycline alone	129	41.48	117	42.24	
Anthracycline plus Taxane	144	46.30	123	44.40	
Taxane alone	20	6.43	19	6.86	
Other	12	3.86	12	4.33	
Adjuvant radiotherapy					0.01
Yes	154	49.52	160	57.76	
No	151	48.55	117	42.24	
Unknown	6	1.93	0	0.00	
Adjuvant endocrine therapy					—
	—		250	90.25	
	—		27	9.75	
(Neo) adjuvant trastuzumab					0.80
Yes	38	12.22	31	11.19	
No	273	87.78	246	88.81	

Abbreviations: LVI: lymphovascular invasion.

**Table 2:** Patients characteristics and treatment pattern in HER2-positive MBC according to HR status.

Characteristic	HR−HER2+		HR+HER2+		*P* ^#^
	*N* = 311	%	*N* = 277	%	
Median age at metastasis(y)	53		50		<0.01
Median DRFI (IQR range) (m)	22.9 (15.3~35.6)		30.7 (18.5–45.9)		<0.01
Median OS follow-up (IQR) (m)	24.8 (14.0~40.3)		32.5 (29.8–46.9)		<0.01
Median No. of metastatic sites (IQR)	2 (1–2)		2 (1–2)		
First-site of distant relapse (%)					
Visceral	207	66.56	165	59.57	0.09
Nonvisceral	104	33.44	112	40.43	
Brain	13	4.18	12	4.33	
Liver	73	23.47	74	26.71	
Lung	110	35.37	68	24.55	
Bone	50	16.08	62	22.38	
Other	65	20.90	61	22.02	
Life-time brain metastasis	79	25.40	68	24.55	0.85
Biopsy for metastatic lesions (%)	45	14.47	39	14.08	0.99
Receptor conversion					
HR+ to HR−	—		11	28.21	
HR− to HR+	4	8.89	—		
HER2+ to HER2−	4	8.89	2	5.13	
HER2− to HER2+	1	2.22	1	2.56	
Trastuzumab-naïve MBC (%)	273	87.78	246	88.81	
Trastuzumab-containing	205	75.09	166	0.67	0.91
first-line	137	66.83	112	67.47	
second-line or beyond	68	33.17	54	32.53	
Median duration of tratuzumab (IQR range) (m)					
	12.0 (5.3~19.6)		16.6 (12.6~29.0)		<0.01
First chemotherapy regimen combined with Trastuzumab*					
Taxane-based	112		100		0.29
Vinorelbine	56		38		
Capecitabine	48		34		
Hormonal therapy	—		10		
Monotherapy	4		2		
Others	6		2		
Median No. of lines of palliative therapy (rang)					
	2 (1~8)		2 (1~8)		

Abbreviations: DRFI, distant recurrence-free interval, IQR, interquartile range.

*Values may add up over 100% because of triple combination was used.

Obviously, HR+HER2+ MBC patients showed a longer DRFI compared with HR−HER2+ patients, with a median DRFI of 30.7 (IQR: 18.5–45.9) months versus 22.9 (IQR: 15.3~35.6) months (*P* < 0.001). Although HR−HER2+ MBC showed a trend towards a higher incidence of visceral metastases (66.5% vs. 59.6%; *P* = 0.09), the difference did not reach statistical significance. The first sites of metastases were similar between HR−HER2+ and HR+HER2+ patients, with brain (4.4% vs. 4.3%), liver (24.6% vs. 26.7%), and lung (35.7% vs. 22.4%) involvement observed. A total of 84 patients (14.3%) received a rebiopsy of metastatic lesions, of which 11 changed from HR-positive to HR-negative, 4 changed from HR-negative to HR-positive, 6 changed from HER2-positive to HER2-negative, and 2 changed from HER2-negative to HER2-positive. However, no robust statistical conclusion could be drawn due to the small number of cases. In terms of above analysis, the most notable clinical feature in HR+HER2+ patients in comparison to HR−HER2+ ones was a significant longer DRFI.

### Determination of the prognostic value of DRFI in HER2+ MBC

**Figure 2:**
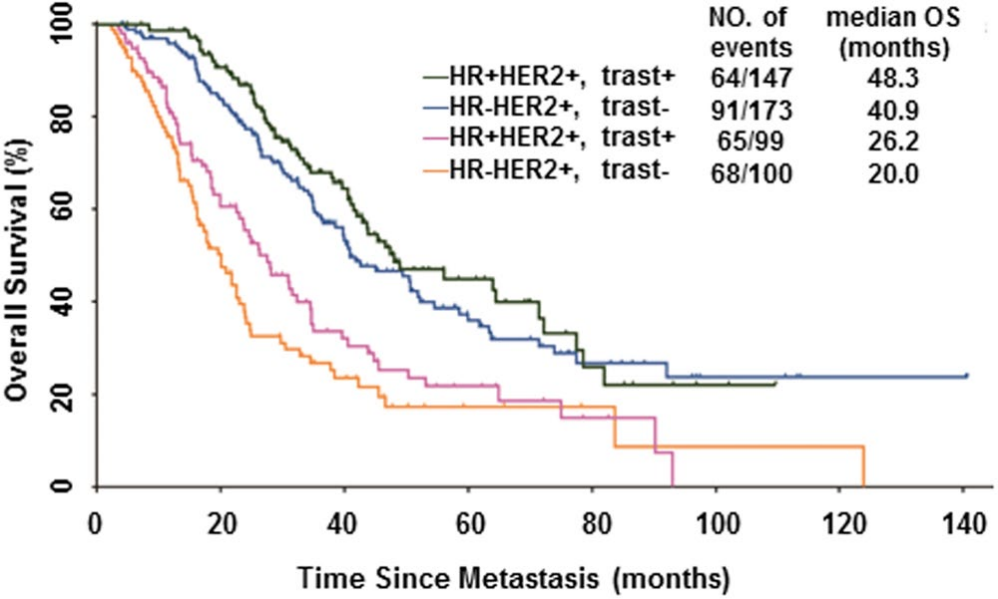
Kaplan-Meier curves of overall survival (OS) in HER2-positive metastatic breast cancer (MBC) patients according to hormone receptor (HR) status and trastuzumab-containing palliative therapy (trast+/−).

Of 511 trastuzumab-naïve MBC patients, 205/273 (75.1%) HR−HER2+ patients and 166/246 (67.4%) HR+HER2+ patients received trastuzumab-containing palliative therapy, with a median trastuzumab treatment duration of 12.0 (IQR: 5.3–19.6) months and 16.6 (IQR: 12.6–29.0) months, respectively. Patients were categorized into four subgroups based on HR status and trastuzumab-containing treatment: HR−HER2+trast+, HR+HER2+trast+, HR−HER2+trast−, and HR+HER2+trast−. The most favourable outcome was observed in the HR+HER2+trast+ subgroup, with a median OS of 48.3 months, compared to 40.9 months in the HR−HER2+trast+ subgroup, 26.2 months in the HR+HER2+trast− subgroup, and 20.0 months in the HR−HER2+trast− subgroup (*P* < 0.0001) (Fig. 2). In total, 137/205 (66.83%) HR−HER2+trast+ patients and 122/166 (67.47%) HR+HER2+trast+ patients received trastuzumab-containing regimens as first-line treatment. The most commonly used co-administered chemotherapy regimens, regardless of HR status, were taxane, vinorelbine and capecitabine. Endocrine therapy combined with trastuzumab as a first-line therapy was only observed in 10/166 (6.02%) HR+HER2+ patients.

The prognostic significance of DRFI after adjustment for potential confounding variables was analyzed, including age, visceral metastasis, number of metastatic sites and palliative trastuzumab therapy. We confirmed that trastuzumab-containing therapy has substantially improved OS both in HR−HER2+ MBC patients (HzR = 0.40, 95% CI: 0.29–0.55, *P* < 0.001) and in HR+HER2+ MBC patients (HzR = 0.41, 95% CI: 0.29–0.58, *P* < 0.001). Multivariate analysis suggested that DRFI > 24 months was an independent favourable prognostic factor for both HR−HER2+trast+ subgroup (HzR = 0.62, 95% CI: 0.40–0.95, *P* = 0.027) and HR+HER2+trast+ subgroup (HzR = 0.59, 95% CI = 0.36–0.97, P = 0.04). In addition, metastatic disease involved ≥2 sites was an independent unfavourable prognostic factor for both HR−HER2+trast+ subgroup (HzR = 1.49, 95% CI: 1.07–2.07, P = 0.018) and HR+HER2+trast+ subgroup (HzR = 1.50, 95% CI = 1.05–2.14, P = 0.025) (Table 3). Collectively, our finding suggested that DRFI was closely related to the biological characteristics of HER2-positive breast tumours, regardless of HR status.

**Table 3:** Univariate and multivariate analysis for overall survival.

Variable	HR−HER2+ (n = 273)						HR+HER2+ (n = 246)					
	Univariate			Multivarites			Univariate			Multivarites		
	HzR	95% CI	P	HzR	95% CI	P	HzR	95% CI	P	HzR	95% CI	P
Age (years)												
≤50	1.48	1.04–2.10	**0**.**030**	1.5	1.04–2.16	**0**.**029**	1.42	1.00–2.03	**0**.**052**			
>50	1	ref						1	ref			
DRFI (months)												
≤24	1	ref					1	ref				
>24	0.64	0.47–0.88	**0**.**006**	0.55	0.39–0.76	**<0**.**001**	0.53	0.38–0.75	**<0**.**001**	0.45	0.32–0.64	**<0**.**001**
NO. of metastatic sites												
isolated	1	ref					1	ref				
≥2	1.31	0.95–1.80	**0**.**094**	1.49	1.07–2.07	**0**.**018**	1.50	1.05–2.14	**0**.**031**	1.50	1.05–2.14	**0**.**025**
First-site of distant relapse												
Nonvisceral	1	ref					1	ref				
Visceral	1.6	1.14–2.25	**0**.**006**	1.51	1.07–2.12	**0**.**019**	1.14	0.81–1.63	0.450			
Palliative-trastuzamb												
trast−	1	ref					1	ref				
trast+	0.39	0.28–0.53	**<0**.**001**	0.4	0.29–0.55	**<0**.**001**	0.43	0.31–0.61	**<0**.**001**	0.41	0.29–0.58	**<0**.**001**

### Interaction of the trastuzumab benefit between DRFI as an continuous variable

**Figure 3:**
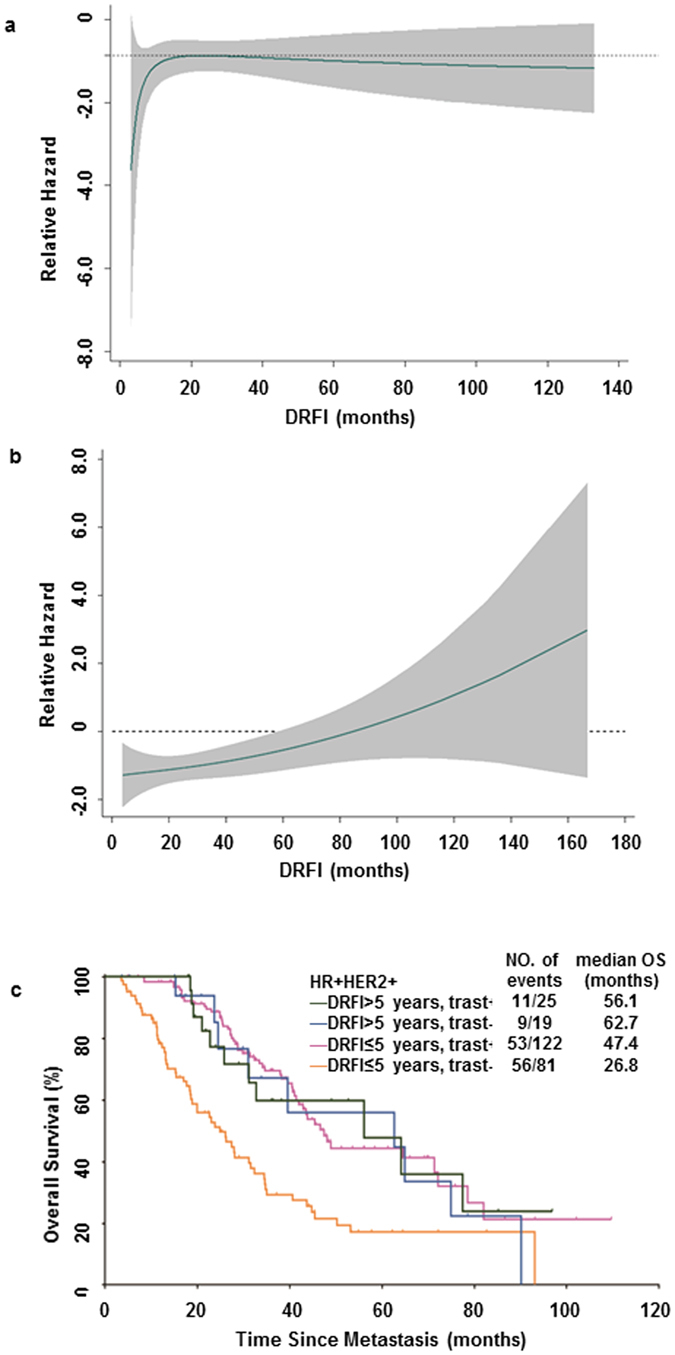
Treatment effect pattern plot showing the interaction between the OS benefit from trastuzumab and DRFI as a continuous covariate. (**a**) For HR−HER2+ MBC, the almost straight line parallel to the x-axis suggests no interaction. (**b**) For HR+HER2+ MBC, the relative hazard ratio was elevated along with DRFI extension. (**c**) The OS curve shows that trastuzumab treatment failed to translate into a survival advantage for later recurrent (DRFI > 5 years) HR+HER2+ MBC patients.

To further investigate the degree of OS benefit, the interaction between degree of trastuzumab benefit and DRFI as a continuous covariate was tested using an multivariable fractional polynomial interaction (MFPI) model^14^. A plot of the log hazard ratio of trastuzumab treatment against the DRFI, together with corresponding 95% CI, is presented in Fig. 3a and b. According to the model, no significant interaction was observed between trastuzumab treatment and DRFI in HR−HER2+ MBC patients, namely, the survival advantage of trastuzumab remained fairly stable up to 6 years of DRFI with a relative hazard ratio less than −0.87 (Fig. 3a). Conversely, for HR+HER2+ MBC patients, the relative hazard ratio kept elevating with prolonged DRFI and reach above zero at DRFI beyond 5 years in trastuzumab treated patients (Fig. 3b). The early recurrent (DRFI ≤ 5 years) HR+HER2+ MBC patients substantially benefit from trastuzumab (HzR = 0.40, 95% CI: 0.27–0.58, *P* < 0.001), whereas the later recurrent (DRFI > 5 years) ones didn’t show similar benefit (HzR = 0.84, 95% CI: 0.32–2.20, *P* = 0.718) (Fig. 3c). Taking into account the possible bias caused by cancer heterogeneity^15^, we further demonstrated the robustness by bootstrap resampling with 10,000 times (Supplementary Figure 1). Our finding suggests that trastuzumab-containing treatment failed to translate into OS benefit in a subgroup of late recurrent HR+HER2+ MBC patients.

## Discussion

Our results confirm that trastuzumab-containing therapy substantially improves OS in both HR+HER2+ and HR−HER2+ MBC patients in China. In accordance with previous reports^16^, we found that HR+HER2+trast+ disease was associated with the most favourable outcome, with a median OS of 48.3 months. Among the prognostic factors investigated, a DRFI > 24 months was independently associated with better OS in HER2-positive MBC regardless of HR status, suggesting that DRFI was closely related to the biological characteristics of both primary tumours and their metastases.

Although there is increasing recognition that HER2-positive MBC is not homogeneous, high-quality prognostic and/or predictive markers with high accuracy and robustness are remain to be identified^15^. The optimization of palliative therapies for HR+HER2+ MBC patients requires further study^8^. Previous reports suggested that high ER expression (≥30%) was associated with a reduced probability of tumour response to trastuzumab plus chemotherapy^11^. However, progression-free survival was significantly improved when maintenance endocrine therapy was added to trastuzumab in ER-positive MBC patients^17^. More importantly, the current analysis provides real-world evidence that DRFI can serve as a reliable stratification factor for OS benefit analysis from trastuzumab-containing palliative therapy in HR+HER2+ MBC, demonstrating that late recurrent (DRFI > 5 years) patients derived a less pronounced survival benefit than their early recurrent counterparts.

Over the last decade, many HER2-positive early breast cancer patients have had limited access to adjuvant trastuzumab worldwide due to reimbursement policies. From January 1, 2007 to December 31, 2011 at FUSCC, only 35.2% (258/732) of HER2-positive primary breast cancer patients received one year of adjuvant trastuzumab^18^, while 83% of such patients in the United States received this treatment. Indeed, the management of trastuzumab-naïve HER2-positive MBC patients will likely continue to be a challenge for physicians. Thus, further study should be carried out to optimize the combination and/or sequence among endocrine therapy, anti-HER2 agents and chemotherapy.

Our study has several limitations. First, we performed a single-institute retrospective database analysis rather than a prospective cohort analysis, which may have led to unaccounted biases. Second, patients who crossed over to receive lapatinib beyond progression were included. Third, adverse effects were not analysed due to unavailable medical records.

In conclusion, our findings reveal that HER2+ metastatic breast cancer can be divided into two distinct subgroups by HR status. HR+HER2+ subgroup have a significant longer DRFI compared to HR−HER2+ subgroup. The interaction between the degree of trastuzumab benefit and DRFI in HR+HER2+ subgroup differed from HR−HER2+ subgroup, demonstrating that DRFI can serve as a reliable stratification factor for the survival benefit from trastuzumab-containing palliative therapy in the HR+HER2+ subgroup.

## Methods

### Ethics statement

Our study was approved by the independent ethical committee/institutional review board of FUSCC. All patients provided written informed consent before inclusion in this study. We obtained permission of FUSCC to collect data from the follow-up database. Our methods were performed in accordance with the relevant guidelines and regulations.

### Patients

This was a retrospective single-institution study conducted at Fudan University Shanghai Cancer Center (FUSCC). Among women (≥18 years old) who were initially diagnosed consecutively with stage I to III breast cancer and received curative surgery and/or radiotherapy at FUSCC from January 1, 2000 to December 31, 2012, 390 subsequently developed distant metastases before December 31, 2013. In addition, HER2-positive MBC patients, who were confirmed by pathology and received palliative therapy in FUSCC from January 1, 2006 to December 31, 2012, were also included (Fig. 1). The following cases were excluded: (1) de novo metastatic disease, (2) bilateral primary breast cancer, (3) inflammatory breast cancer, (4) second primary invasive cancer, and (4) prior lapatinib exposure before trastuzumab.

The following data were collected: age at initial diagnosis, menopausal status, date of diagnosis of primary breast cancer, histological type, size of primary tumour, number of positive lymph nodes, oestrogen receptor (ER) and progesterone receptor (PgR) status, HER2 status (including immunohistochemistry (IHC) results and fluorescent *in situ* hybridization (FISH) results if available), Ki67 index, date of metastasis, sites of metastases, receptor status of metastatic lesion (if reassessed), and date of death. The treatment history was also collected, including prior (neo)adjuvant chemotherapy, (neo)adjuvant endocrine therapy, and (neo)adjuvant anti-HER2 therapy. The dates of the first and last courses of trastuzumab were also recorded.

Positivity of ER and PgR status was defined as immunostaining ≥1% of invasive tumour cells. HER2 positivity was defined as neu staining of 3+ by immunohistochemistry or 2+ with gene amplification by FISH. Patients were considered to have visceral disease if they had one of the following organs involved: liver, lung, pleura, adrenal gland, pericardial/peritoneal cavity, spleen, thyroid or brain. For patients who developed multi-organ metastasis, the first site of metastasis was ranked according to the prognosis: brain, liver, lung, bone, and other (e.g., lymph node, soft tissue, skin, pleura, and other rarer sites).

### Statistical analysis

DRFI was defined as the interval between the diagnosis of primary breast cancer and the diagnosis of the first distant relapse^19^. OS was defined from the diagnosis of distant metastatic disease to the date of death from any cause. Follow-up was conducted by specific staff members until March, 31, 2016. If patients were lost during the follow-up period, the data were censored at the last date recorded.

The patient demographics and tumour characteristics by HR status were compared using the using Chi-square and the t test. Kaplan-Meier curves for overall survival rates were created using GraphPad Prism (Version 4.03). Univariate regression between variables and overall survival were evaluated. All variables associated with P < 0.10 on univariate analysis were included in multivariate Cox proportional hazards model. Hazard ratios (HzR) with 95% confidence intervals were tested for all entered variables with backward method (SPSS Version 20, IBM Corporation, Armonk, NY, USA). The interaction between trastuzumab benefit and DRFI was tested with multivariable fractional polynomial interaction (MFPI) using Stata (Version 13)^14^. All statistical tests were two-sided, and a *P*-value < 0.05 was considered significant.

## Electronic supplementary material

Supplementary Figure 1
