# Author Correction: Impact of hormone receptor status and distant recurrence-free interval on survival benefits from trastuzumab in HER2-positive metastatic breast cancer

**DOI:** 10.1038/s41598-018-32881-6

**Published:** 2018-10-12

**Authors:** Hai-Yuan Yang, Ding Ma, Yi-Rong Liu, Xin Hu, Jian Zhang, Zhong-Hua Wang, Gen-Hong Di, Xi-Chun Hu, Zhi-Ming Shao

**Affiliations:** 1 Department of Breast Surgery, Fudan University Shanghai Cancer Center, Department of Oncology, Shanghai Medical College, Fudan University, Shanghai, 200032 China; 2 Department of Medical oncology, Fudan University Shanghai Cancer Center, Department of Oncology, Shanghai Medical College, Fudan University, Shanghai, 200032 China

Correction to: *Scientific Reports* 10.1038/s41598-017-00663-1, published online 25 April 2017

In Figure 2 the subgroup order is incorrect.

**Figure 2:**
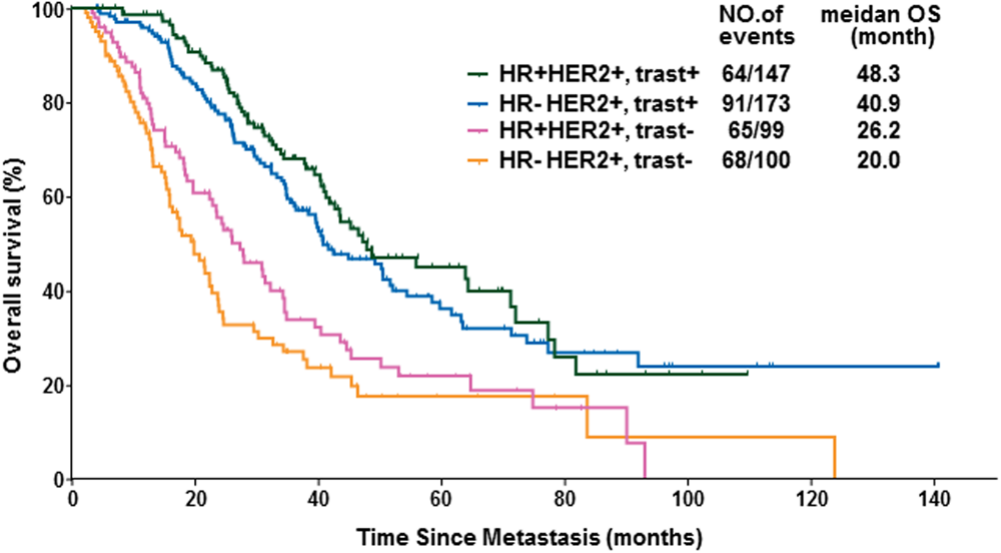
Kaplan-Meier curves of overall survival (OS) in HER2-positive metastatic breast cancer (MBC) patients according to hormone receptor (HR) status and trastuzumab-containing palliative therapy (trast+/−).

HR+HER2+, trast+

HR−HER2+, trast−

HR+HER2+, trast+

HR−HER2+, trast−

should read:

HR+HER2+, trast+

HR−HER2+, trast+

HR+HER2+, trast−

HR-HER2+, trast−

The correct Figure appears below.

