# Supplementary material for: Impact of hormone receptor status and distant recurrence-free interval on survival benefits from trastuzumab in HER2-positive metastatic breast cancer
Source: Sci Rep. 2017 Apr 25;7:1134. doi: 10.1038/s41598-017-00663-1 (PMC5430907; doi:10.1038/s41598-017-00663-1)

Impact of hormone receptor status and distant recurrence-free interval on survival benefits from trastuzumab in HER2-positive metastatic breast cancer

Hai-Yuan Yang[1], Yi-Rong Liu[1], Ding Ma[1], Xin Hu[1], Jian Zhang[2], Zhong-Hua Wang[2], Geng-Hong Di[1*], Xi-Chun Hu[2], Zhi-Ming Shao[1]

Supplementary Figure 1. Bootstrap resampling with 10,000 times verified interaction between the OS benefit and DRFI as a continuous covariate. Red dashed lines: 95%CI estimated using 10,000 bootstrap samples; Blue line: original data; Blue dashed lines: 95%CI estimated using original data; Grey lines: first 1000 bootstrap samples.

a

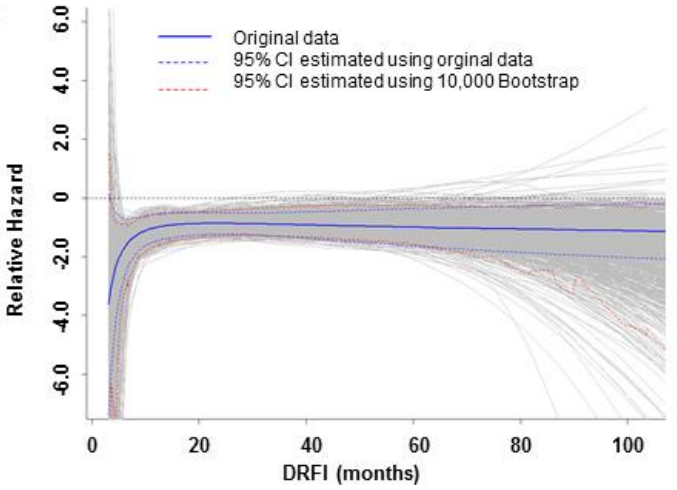

b

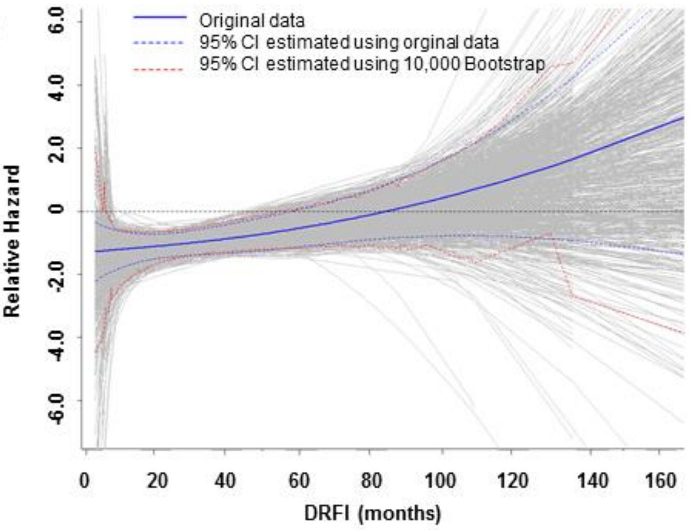

Supplement: Supplementary file 1 — Supplementary Figure 1 [file 41598_2017_663_MOESM1_ESM.pdf]
